# Effects of Low Temperature, Nitrogen Starvation and Their Combination on the Photosynthesis and Metabolites of *Thermosynechococcus* E542: A Comparison Study

**DOI:** 10.3390/plants10102101

**Published:** 2021-10-04

**Authors:** Xingkang Li, Yuanmei Liang, Kai Li, Peng Jin, Jie Tang, Anna Klepacz-Smółka, Stanislaw Ledakowicz, Maurycy Daroch

**Affiliations:** 1 School of Environment and Energy, Peking University Shenzhen Graduate School, Shenzhen 518055, China; xkli@gzu.edu.cn (X.L.); yuanmeiliang@pku.edu.cn (Y.L.); likai0513@pku.edu.cn (K.L.); jinpeng@pkusz.edu.cn (P.J.); 2 Department School of Liquor and Food Engineering, Guizhou University, Guiyang 550025, China; 3 School of Food and Bioengineering, Chengdu University, Chengdu 610106, China; tangjie@cdu.edu.cn; 4 Department of Bioprocess Engineering, Faculty of Process and Environmental Engineering, Lodz University of Technology, ul. Wolczanska 213, 90-924 Lodz, Poland; anna.klepacz-smolka@p.lodz.pl (A.K.-S.); stanislaw.ledakowicz@p.lodz.pl (S.L.)

**Keywords:** cyanobacteria, chlorophyll fluorescence, alkane, lipidomics, carbohydrate, nitrogen starvation, low temperature

## Abstract

Both low temperature and nitrogen starvation caused chlorosis of cyanobacteria. Here, in this study, for the first time, we compared the effects of low temperature, nitrogen starvation, and their combination on the photosynthesis and metabolites of a thermophilic cyanobacterium strain, *Thermosynechococcus* E542. Under various culture conditions, the growth rates, pigment contents, and chlorophyll fluorescence were monitored, and the composition of alkanes, lipidomes, and carbohydrates were determined. It was found that low temperature (35 °C) significantly suppressed the growth of *Thermosynechococcus* E542. Nitrogen starvation at 45 °C and 55 °C did not affect the growth; however, combined treatment of low temperature and nitrogen starvation led to the lowest growth rate and biomass productivity. Both low temperature and nitrogen starvation caused significantly declined contents of pigments, but they resulted in a different effect on the OJIP curves, and their combination led to the lowest pigment contents. The composition of fatty acids and alkanes was altered upon low-temperature cultivation, while nitrogen starvation caused reduced contents of all lipids. The low temperature did not affect carbohydrate contents, while nitrogen starvation greatly enhanced carbohydrate content, and their combination did not enhance carbohydrate content, but led to reduced productivity. These results revealed the influence of low temperature, nitrogen starvation, and their combined treatment for the accumulation of phycobiliproteins, lipids, and carbohydrates of a thermophilic cyanobacterium strain, *Thermosynechococcus* E542.

## 1. Introduction

An increase in CO_2_ concentration in the atmosphere caused by the exploitation of fossil fuels results in global climate change and threatens the sustainable development of humankind. Carbon sequestration technologies have attracted the wide attention of both academics and industries. Cyanobacteria are prokaryotic photosynthetic micro-organisms able to inhabit various environments and tolerate different stresses, such as high salinity, temperature, pressure, and desiccation [1]. Thanks to their high photosynthetic rates and potential to produce various value-added products and biofuels, cyanobacteria have been promising candidates for biological carbon sequestration [2,3].

Growth temperature and nitrogen supply are two major factors that regulate the photosynthetic rates and biochemical composition of cyanobacteria [4,5]. Mesophilic cyanobacteria acclimate to low temperature (temperatures 15–20 °C lower than their optimal growth temperature), either by rearrangement of their photosynthetic system on a short time scale [6] or by reduction in phycobiliprotein contents on a longer time scale [7], which could be reflected by chlorophyll fluorescence analysis. The chemical composition also changed with growth temperatures, such as the ratio of unsaturated fatty acids to saturated fatty acids and the contents of protein and carbohydrates [8]. In response to nitrogen starvation, nondiazotrophic cyanobacteria tend to synthesise fewer components rich in N, such as proteins, chlorophylls, and phycobilisomes (PBSs). Nitrogen starvation also caused the accumulation of compounds rich in C, such as glycogen [9].

Although numerous studies have been focused on the strategies cyanobacteria adopted to acclimate to low temperature or nitrogen starvation, no study has compared the effects of low temperature, nitrogen starvation, and, especially, their combined effects on photosynthesis and biochemical composition. It was reported that the dependence of metabolic rates of phytoplankton (*Synechococcus* sp., *Skeletonema costatum*, and *Emiliania huxleyi*) on growth temperature were strongly related to nitrogen supply [10]. Additionally, previous studies on the acclimation of cyanobacteria to growth temperature and nitrogen starvation focused mainly on mesophilic strains, while there is a scarcity of similar studies regarding thermophilic strains. Thermophilic strains have recently been suggested as thermophilic chassis for carbon valorisation, which could significantly reduce the cost of CO_2_ mitigation from flue gases [11,12]. Previously, we have described a new thermophilic cyanobacterium, *Thermosynechococcus elongatus* PKUAC-SCTE542 (*Thermosynechococcus* E542 since), isolated from the hot spring of Western Sichuan, China as a promising organism for CO_2_ utilisation [11]. The goal of this study was to characterise and compare the effects of low temperature, nitrogen starvation, and their combination on photosynthesis by analysis of chlorophyll-*a* (Chl*a*) fluorescence and pigments, and to analyse its accumulation of main components, including lipids, proteins, and carbohydrates.

## 2. Results

### 2.1. Effects of Temperature, Nitrogen Starvation, and Their Combination on the Growth

A two-stage culture strategy was adopted to investigate the effects of growth temperature and nitrogen supply. In the first stage, *Thermosynechococcus* E542 was cultivated for 24 h in BG-11 medium supplemented with sodium nitrate. At the end of the first stage, the biomass was collected by centrifugation and then resuspended in a fresh BG-11 medium supplemented with or without sodium nitrate. It was then incubated for another 24 h (the second stage), and the results related to the growth rate and biomass yield are shown in Table 1.

Growth temperature significantly affected the growth rate and biomass yield of *Thermosynechococcus* E542. During the first 24 h, when temperature declined from 45 °C to 35 °C, the growth rate was reduced by 42.9% from 2.26 to 1.29 d^−1^, and the 48-h average biomass productivity was increased by 123% from 0.132 g L^−1^ d^−1^ to 0.296 g L^−1^d^−1^ when growth temperature was elevated from 35 °C to 45 °C. The effect of temperature on growth rate became less pronounced when it was further increased from 45 °C to 55 °C. The growth rate was relatively constant, confirming prior findings [11].

Intriguingly, it was found that nitrogen starvation did not affect the growth rate and biomass productivity at 45 °C and 55 °C. However, combined treatment of nitrogen starvation and low temperature at 35 °C caused the growth rate to decline by 20.7% from 0.82 to 0.65, while biomass productivity also declined from 0.148 g L^−1^d^−1^ to 0.094 g L^−1^d^−1^.

### 2.2. Variation of Pigment Contents and Chlorophyll Fluorescence

**Figure 1:**
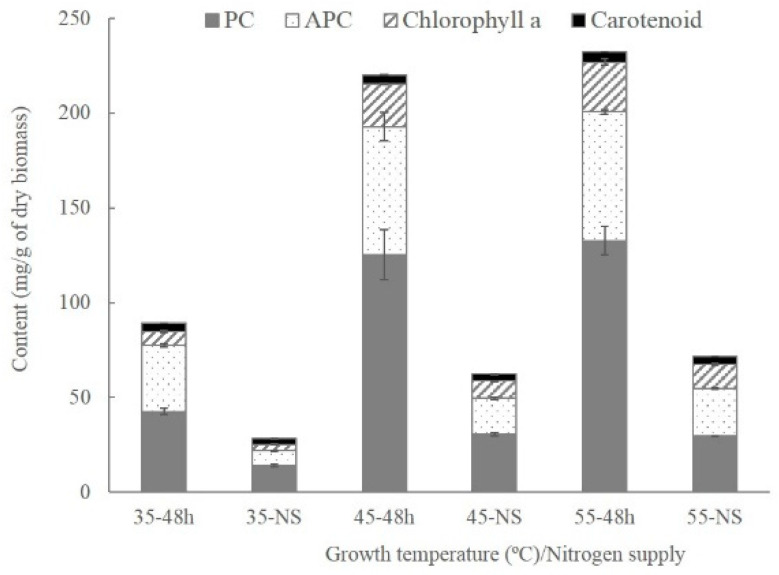
Contents of pigments of E542 cultivated under different conditions. Bars indicate standard deviation; data are expressed as means ± standard error (n = 3).

*Thermosynechococcus* E542 cultivated at 35 °C exhibited lower contents of Chl*a* and PC. The cultures that were grown at 45 °C and 55 °C in nitrogen-rich BG-11 medium contained an abundance of pigments, C-phycocyanin (PC) and allophycocyanin (APC) accounted for 12–13% and 6.8–6.8% of dry biomass, respectively, while Chl*a* reached more than 2% of the biomass (Figure 1). However, at 35 °C, their content was dramatically reduced by 66.1% and 67.4%, respectively, compared with cultures grown at 45 °C. The ratio of PC to Chl*a* did not decline with decreasing growth temperature and was estimated at 5.8, 5.6, and 5.1, respectively, for growth temperatures of 35 °C, 45 °C, and 55 °C. These findings are in contrast to previous research that indicates that PC/Chl*a* is reduced during low-temperature acclimation of mesophilic cyanobacteria strains [13].

Nitrogen starvation treatment led to significantly reduced pigment contents in *Thermosynechococcus* E542 at all tested temperatures. The content of PC was 67–78% lower, while the content of Chl*a* was 51–60% lower than cultures grown in nitrogen-rich BG-11 medium. Combined treatment of low temperature (35 °C) and nitrogen starvation led to the lowest pigment contents among all conditions. Contents of PC, APC, and Chl*a* declined to 1.4%, 0.78%, and 0.34% of dry biomass.

**Figure 2:**
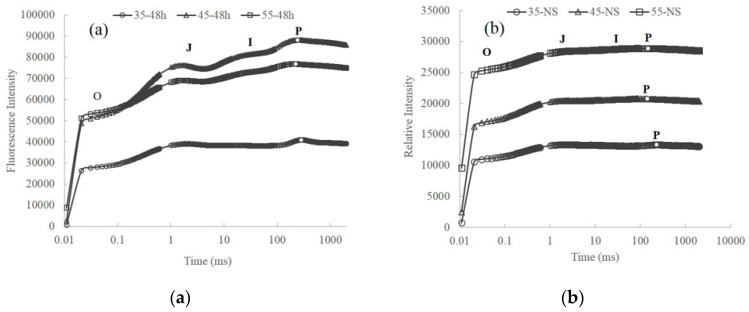
The OJIP fluorescence transients of E542 cultivated at different temperatures (**a**) in nitrogen-rich BG-11 medium; (**b**) with nitrogen starvation. The transient is plotted on the logarithmic time scale, and the O, J, and I steps are marked accordingly, while the P step is marked by dots on the curves. All experiments were conducted with three replicas, and the means were used to construct the figure.

Growth temperature significantly affects the OJIP curves of *Thermosynechococcus* E542. Figure 2a shows the fluorescence transient curves of *Thermosynechococcus* E542 cultivated at different temperatures in a nitrogen-rich BG-11 medium. The transient curves showed clear polyphasic rise, with the I and J step appearing at 2 ms and 30 ms, respectively, while the P step appeared at 141-281 ms. Significant differences between the OJIP transients for the culture 35–48 h and those of 45–48 h and 55–48 h could be observed. Firstly, the culture 35–48 h has much lower F*_O_* fluorescence, 44.6% lower than that of 45–48 h. Similar results were obtained for eukaryotic photosynthetic organisms and mesophilic cyanobacterium *Synechococcus* sp. PCC 7942 [14]. F*_O_* fluorescence in cyanobacteria considerably depends on the cellular phycobilin concentration [15]. In this study, OD_730_ of all cultures was adjusted to the same value (0.26 ± 0.01) prior to the measurement of OJIP transients. Hence, the lower F*_O_* fluorescence of 35–48 h was due to its lower PC content. Another difference is that the relative variable fluorescence at step J (V_J_ = (F_J_ − F*_o_*)/(F_m_ − F*_o_*)) of the culture 35–48 h is much higher than that of 45–48 h and 55–48 h (Table 2). This indicates that the reoxidation capacity of Q_A_^−^ is decreased at a growth temperature of 35 °C [16]. Growth temperature also affects the photochemical efficiency of PSII in the dark-adapted state (F_v_/F_m_). F_v_/F_m_ reaches the maximal value of 0.422 at 45 °C, close to the maximal values of 0.4–0.5 for mesophilic cyanobacteria [6].

Nitrogen starvation also affected the shape of the OJIP transients of *Thermosynechococcus* E542, as shown in Figure 2b. Due to the degradation of pigments, the F*_O_* fluorescence declined to 10,000–15,000 after nitrogen starvation, compared with 25,000–50,000 for cultures replete in nitrogen. Additionally, the OJIP transients almost totally level off after nitrogen starvation treatment. Similar results were observed for the OJIP transients of mesophilic cyanobacteria under other stress factors, such as high salinity [17] and heat stress [18]. Moreover, the photochemical efficiency of PSII also sharply declined to 0.18–0.20 from 0.3–0.4 (Table 2).

### 2.3. Variation of Alkanes and Fatty Acids

**Figure 3:**
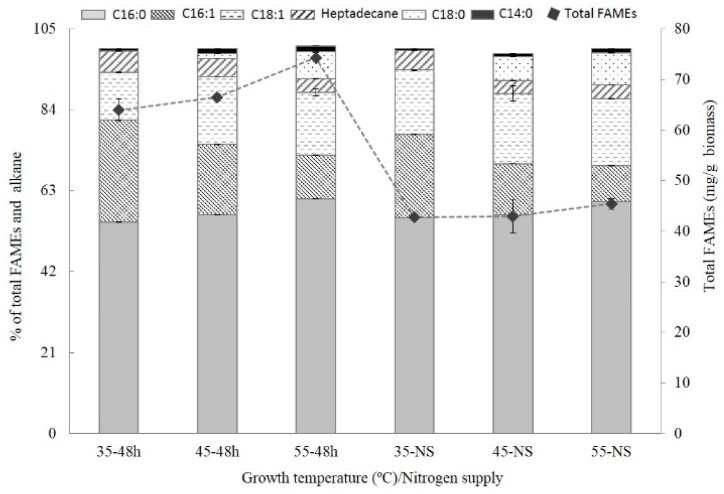
Effects of temperature and nitrogen starvation on the contents of alkane and FAMEs. Bars indicate standard deviation; data are expressed as means ± standard error (n = 3).

Membrane fluidity plays a pivotal role in the temperature acclimation of photosynthetic organisms [8]. Adjustment of the membrane fluidity could be achieved by the production of polyenoic acid or by changing the ratio of saturated fatty acids to unsaturated fatty acids (S/U ratio) in mesophilic cyanobacteria [19]. Recently, it was found that hydrocarbons may also be involved in the adjustment of membrane fluidity during low-temperature acclimation of cyanobacteria [20]. The composition of fatty acids and hydrocarbons of *Thermosynechococcus* E542 cultivated at different temperatures and nitrogen supply is shown in Figure 3. Under the tested conditions, palmitic acid (C16:0) is the most abundant fatty acid, accounting for 55–60% of total fatty acid methyl esters (FAMEs) and hydrocarbons. Other fatty acids include palmitoleic acid (C16:1), stearic acid (C18:0), oleic acid (C18:1), and myristic acid (C14:0). The hydrocarbons include pentadecane and heptadecane, but the amount of pentadecane is not quantitated due to its limited concentration.

Growth temperature significantly affects the composition of fatty acids and hydrocarbons. The content of C16:1 is inversely proportional to the growth temperature and increased by 61% and 135% as growth temperature decreased from 55 °C to 45 °C and 35 °C, respectively. S/U ratio was also altered and was equal to 1.4, 1.7, and 2.5 at the growth temperatures of 35 °C, 45 °C, and 55 °C, respectively. The content of heptadecane also depends on growth temperature. As growth temperature decreased from 55 °C to 45 °C and 35 °C, the amount of heptadecane increased by 25% and 49%, respectively, suggesting an increase in membrane fluidity.

Nitrogen starvation caused a significant decline in the total amount of FAMEs and hydrocarbons from 6–7% to about 4%. However, nitrogen starvation did not affect the correlation between FAMEs and hydrocarbon composition and growth temperature. This is consistent with previous findings that nitrogen starvation caused the degradation of the intracellular membranes of Synechococcus elongates PCC 7942 [9].

### 2.4. Lipidomic Analysis Based on LC-MS/MS

In total, 56 polar glycerides in *Thermosynechococcus E542* were identified, including 29 monogalactosyldiacylglycerols (MGDG), 20 digalactosyldiacylglycerols (DGDG), 3 sulfoquinovosyl-diacylglycerols (SQDG), and 4 phosphatidylglycerols (PG) (Table S1). To the best of our knowledge, no prior reports on the lipidome of thermophilic cyanobacteria based on LC-MS/MS have been found in the literature. Besides, our results showed that *Thermosynechococcus E542* could synthesise glycerides containing fatty acids with an odd number of carbon atoms, such as C17:0 and C19:1. Glycerides containing odd-number fatty acids were also observed in other cyanobacteria strains. Glycerides containing C17:1, C17:2, and C17:3 were detected by two-dimensional liquid chromatography with quadrupole time-of-flight mass spectrometry in *Synechococcus* sp. PCC 7002 [21]. Glycerides containing C17:1 and C17:2 were identified in *Synechocystis sp. PCC 6803* by easy ambient sonic-spray ionisation mass spectrometry [22].

DGDG and MGDG were the most abundant polar lipids in *Thermosynechococcus E542*, accounting for 54 ±1.6% and 37 ± 1.5 % of total polar lipids, respectively. The contents of SQDG and PG were lower. Growth temperature did not significantly affect the absolute amount of various polar lipids; the contents of DGDG, MGDG, PG, and SQDG were quite close at all tested temperatures with the same nitrogen supply (Table 3). In addition, the ratio of different lipids to total polar lipids remained stable under different culture conditions. Pittera et al. studied the effect of culture temperature on the lipidome of marine cyanobacteria *Synechocuccus* sp. WH7803. It was shown that the ratio of MGDG, DGDG, SQDG, and PG remained stable at different temperatures, accounting for 45.4 ± 4.1%, 23.0 ± 3.6%, 24.1 ± 3.9%, and 7.5 ± 2.3% of total polar lipids, respectively [23]. The composition of fatty acids in DGDG and MGDG changed with culture temperature, regardless of nitrate supply (Table 3). The main DGDG species were DGDG (16:0/18:1), DGDG (16:0/16:1), and DGDG (16:0/16:0), and these species accounted for more than 70% of DGDG. As growth temperature decreased from 55 °C to 35 °C, the content of DGDG (16:0/16:1) substantially increased from 19–25% to 50–60%, while the contents of DGDG (16:0/16:0) and DGDG (16:0/18:1) significantly decreased. A similar pattern was observed for MGDG. At 55 °C, MGDG (16:0/16:1) accounted for 21–27% of total MGDG, while, at 35 °C, its content greatly increased to 47–64 %. Three SQDG species were detected in *Thermosynechococcus E542*. Under all tested culture conditions, SQDG (16:0/16:0) accounted for more than 99% of all SQDG species, which suggested that growth temperature and nitrogen supply did not affect the structure of fatty acids in SQDG. Growth temperature also affected the structure of fatty acids in PG, but the pattern was different than that of DGDG and MGDG. At 55 °C, the main PG species were PG (16:0/16:0), PG (16:0/16:1), and PG (18:0/16:0), which accounted for 18–19%, 62–66%, and 16–19% of total PG, while PG (16:0/18:1) was not detected. As growth temperature decreased to 35 °C, the content of PG (16:0/18:1) sharply increased from 0 to more than 40%, while the content of PG (18:0/16:0) decreased from 16–19% to 0.

Nitrogen supply had a significant effect on the contents of various lipids at all tested temperatures. With nitrate depleted, at 35 °C, the contents of MGDG, DGDG, SQDG, and PG were reduced by 31%, 46%, 53%, and 35%, respectively, compared with nitrate-repleted culture, while, at 55 °C, they were reduced by 26%, 16%, 39 %, and 41%, respectively. Besides, the structure of fatty acids in specific lipids was also changed. However, these patterns were not as clear as those observed for temperature changes.

### 2.5. Content and Productivity of Carbohydrate and Elemental Composition of the Biomass

**Figure 4:**
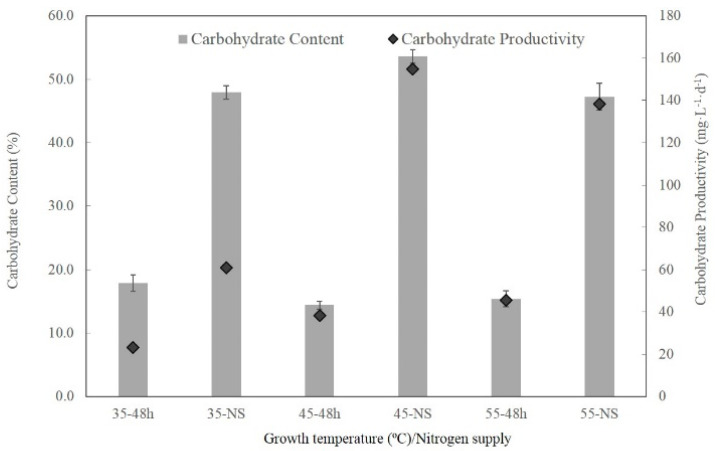
Effects of temperature and nitrogen starvation on the content and productivity of glycogen. Bars indicate standard deviation; data are expressed as means ± standard error (n = 3).

Growth temperature did not affect the content of carbohydrates significantly. As shown in Figure 4, the samples cultivated in nitrogen-rich BG-11 medium contain only 14–17% of carbohydrates, leading to low carbohydrate productivity of 20–40 mg L^−1^d^−1^. Elemental analysis shows that nitrogen accounts for 8–9% of the dry weight (Table 4), indicating that the biomass comprises more than 50% protein (calculated using a conversion factor of 6.25 from nitrogen to protein). Nitrogen starvation significantly promoted the content and productivity of carbohydrates. Maximal carbohydrate content and carbohydrate productivity were obtained at 45 °C, which was 53.7% and 154 mg L^−1^d^−1^, respectively. Meanwhile, the nitrogen content in the biomass declined by more than 50% from 8–9% to about 4%. Combined treatment of low temperature and nitrogen starvation did not improve carbohydrate content and carbohydrate productivity.

## 3. Discussion

Growth of *Thermosynechococcus* E542 at 35 °C, 20 °C lower than the optimal growth temperature, led to significantly lower growth rate, biomass productivity, and pigment contents. This is in accordance with the previous study suggesting that photoinhibition of mesophilic cyanobacteria usually occurred at 15 °C lower than their optimal temperature [6]. In addition, the abundance of pigments in mesophilic cyanobacteria could be drastically altered during acclimation to low temperatures. Vonshak et al. found that, when *Arthrospira platensis* strains M_2_ and Kenya were cultivated at 15 °C, the contents of Chl*a* and PC were reduced by 59.6% and 60.9%, 56.5%, and 68.2%, respectively, compared with those at 30 °C [13].

Nitrogen starvation also caused a significant decline in PBSs and Chl*a* in non-diazotrophic mesophilic cyanobacteria [4]. In line with these results, it was found that pigment contents of *Thermosynechococcus* E542 were also reduced upon nitrogen starvation. Upon nitrogen depletion, not only is synthesis of PBS in cyanobacteria depressed, but, also, rapid degradation of PBS occurs. Nutrient starvation induced the expression of *nblA* gene, which is essential for PBS degradation in cyanobacteria [24]. Our previous study confirmed the existence of *nblA* gene in the genome of *Thermosynechococcus* E542. It was found that *nblA* is responsible for cell bleaching upon nutrient depletion [11].

Nitrogen starvation led to reduced contents of pigments at all tested growth temperatures. However, at 45 °C and 55 °C, 24-h nitrogen starvation did not affect the growth of *Thermosynechococcus* E542. This suggests that a considerable proportion of pigments (more than 50%) accumulated during growth in the nitrogen-rich medium can serve as a nitrogen source during growth in the nitrogen-depleted medium. These findings are in accordance with previous studies based on mesophilic cyanobacteria *Synechococcus* sp. PCC 7942 [25] and *Synechococcus* sp. PCC 6803 [26]. Although growth at 35 °C also led to reduced pigment contents, they were higher than those observed under nitrogen starvation treatment. However, growth rate and biomass productivity were significantly lower when cultivated at 35 °C than at 45 °C and 55 °C. This indicates that the decline in pigments should not be the cause of reduced growth rate and biomass productivity. Combined treatment of nitrogen starvation and low temperature caused the lowest growth rate, biomass productivity, and pigment contents, suggesting that low temperature acclimation of *Thermosynechococcus* E542 depends on nitrogen supply. Analysis of Chl*a* fluorescence showed that the impact of nitrogen starvation and low growth temperature on the electron transport chain are different, although both treatments caused reduced pigment contents. The OJIP curves showed typical multiphasic rises at all growth temperatures without nitrogen starvation, but almost totally levelled off after 24-h nitrogen starvation.

The effect of nitrogen starvation and low temperature on the composition of fatty acids and hydrocarbons was also different. During low-temperature acclimation, the ratio of saturated fatty acids to unsaturated fatty acids and the content of heptadecane increased, but the total amount of fatty acids and hydrocarbons was maintained constant. In comparison, after 24-h nitrogen starvation, the total amount of fatty acids and hydrocarbons decreased. Analysis of the lipidome based on LC-MS/MS showed that growth temperature did not affect the absolute amount of various lipids in the biomass. However, the structure of fatty acids in lipids was altered, although in different patterns for different lipid classes. For MGDG and DGDG, species with a fatty acid composition of 16:0/16:1 increased with decreasing temperature. In addition, for PG, the most significant change was that PG (16:0/18:1) sharply increased with reduced temperature. On the other hand, the composition of fatty acids in SQDG was not affected by temperature. In comparison, nitrate depletion caused a significant decline in the absolute contents of various lipids. In contrast, the change in fatty acid structure in specific lipids was not as apparent as observed for temperature.

Low temperature and nitrogen starvation also had different impacts on the content and productivity of carbohydrates. The low temperature did not increase the content of carbohydrates but significantly reduced their productivity. However, the content and productivity of carbohydrates were greatly enhanced upon nitrogen starvation. The content and productivity of carbohydrates were higher than those of mesophilic cyanobacteria strains *Synechocystis* sp. PCC6803 [26] and *Synechococcus elongatus* PCC7942 [27].

## 4. Materials and Methods

### 4.1. Cultivation of Cyanobacterium

Unless otherwise stated, cyanobacterium *Thermosynechococcus elongatus* PKUAC-SCTE542 (hereafter, *Thermosynechococcus* E542 in abbreviation) deposited in the Freshwater Algae Culture Collection at the Institute of Hydrobiology (FACHB-2455) was cultivated in BG-11 medium supplemented with 0.1 M NaHCO_3_, prepared by mixing one portion of twofold BG-11 and one portion of 0.2 M solution of NaHCO_3_ in equal volumes. In nitrogen starvation experiments, NaNO_3_ free BG-11 (BG11-N hereafter) was used instead of standard BG-11. The strain was cultivated in a shaking incubator (Bluepard, Yiheng Group, Shanghai, China) at 35, 45, or 55 °C, 120 rpm. The shaking incubator was modified with white LED strips to provide two-sided LED lighting. Photosynthetic photon flux density was 109 μmol m^−2^s^−1^ (measured with Lighting Passport, Asense Tek, Tainan, China). The light/dark cycle was 23/1 for all experiments. Samples from precultures were diluted in 150 mL fresh BG-11 medium in 500 mL Erlenmeyer flasks to OD_730_ of 0.12 ± 0.1, corresponding to 0.027 gL^−1^. After 24 h of incubation, the biomass was collected by centrifugation at 10,000 rpm for 3 min and resuspended in fresh BG-11 or BG-11-N (for nitrogen starvation experiments), supplemented with 0.1 M NaHCO_3_, and incubated for another 24 h. All experiments were conducted in three biological replicas. At specific times, an aliquot of the culture was withdrawn and OD_730_ was determined (UV-1800, Shimadzu Corporation, Kyoto, Japan). The culture was diluted by fresh medium if OD_730_ exceeded 1.0. At 48 h, the biomass was collected by centrifugation at 10,000 rpm for 3 min, washed with 30 mL deionised water, and freeze-dried for 48 h at −50 °C, 12 Pa (Freezone 2.5, Labcon, USA). Specific growth rates (μ, d^−1^) were determined using biomass concentration (gL^−1^) at the beginning and end of the exponential growth phase using Equation (1).

(1) $$\mu \;=\left({\mathrm{lnX}}_{2}-{\mathrm{lnX}}_{1}\right)\;/\;\left(t_{2}-t_{1}\right)$$

where X_1_ and X_2_ are biomass concentration at times t_1_ and t_2_, respectively.

### 4.2. Chla Fluorescence Transient Analysis

The fluorescence intensity of photosynthetic organisms shows polyphasic increases upon dark to light transition from a low level of F*_O_* via two intermediates F_J_ and F_I_ to a maximum level of F_M_. Each rise phase can be related to a specific step of the reduction in the ETC, and thus information on the state of the ETC can be obtained via analysis of the OJIP transients. In general, the OJ step is related to the reduction in Q_A_ to Q_A_^−^ and, hence, the accumulation of Q_A_^−^ Q_B_, the JI step is related to the accumulation of Q_A_^−^ Q_B_^−^, and the IP step reflects an accumulation of Q_A_^−^ Q_B_^2−^ [16].

Polyphasic rise of Chl*a* fluorescence transient (OJIP) measurements were conducted using pulse amplitude magnitude (PAM) fluorimeter (AquaPen-C AP-C 100, Photon Systems Instruments, Czech Republic) and FluorPen 1.0 software. All samples were diluted with the same medium of the culture OD_730_ of 0.26 ± 0.01 and were incubated in the dark for at least 15 min before measurements. Fluorescence was recorded at the same temperature as the culture by housing the fluorimeter and the sample in the incubator. Red-orange light (620 nm) of 3000 μmol m^−2^s^−1^ was used for saturating light pulses in the fluorimeter. The PAM fluorimeter recorded the Chl*a* fluorescence induced by the saturating pulses between 20 μs and 1 s. The fluorescence intensity at 50 μs, 2 ms, and 30 ms was designated as O, J, and I fluorescence, respectively, whilst P was the maximum fluorescence. For more details about the procedure and parameters of OJIP, please refer to [28].

### 4.3. Measurement of Pigments

To determine Chl*a* and carotenoids, 4 mL aliquot was withdrawn from the culture and transferred to a 15 mL centrifuge tube and centrifuged at 15,000× *g* for 7 min. The supernatant was discarded and 4 mL methanol precooled to 4 °C was added [29]. The centrifuge tube was covered with aluminium foil and incubated at 4 °C for 20 min. After being centrifuged at 15,000× *g* for 7 min, Chl*a* and carotenoids dissolved in the supernatant were determined by measuring optical absorbance at 470, 665, and 720 nm using methanol as blank (UV-1800, Shimadzu Corporation, Kyoto, Japan). Concentrations of Chl*a* and carotenoids were calculated according to the following equations [30]:

(2) $$\mathrm{Chl}a\;\left[\mu g/\mathrm{mL}\right]=12.9447\times \left(A_{665}-A_{720}\right)$$

(3) Chla [μg/mg of biomass]= Chla[μg/mL] / biomass concentration[mg/mL]

(4) $$\mathrm{Carotenoids}\;\left[\mu g/\mathrm{mL}\right]=\left[1000\times \left(A_{470}-A_{720}\right)-2.86\times \left(\mathrm{Chl}a\left[\mu g/\mathrm{mL}\right]\right)\right]/221\;$$

(5) Chla [μg/mg of biomass]= Chla[μg/mL] / biomass concentration[mg/mL]

To determine phycobiliprotein, 1.5 mL culture suspension was transferred to centrifuge tubes and was centrifuged at 15,000× *g* at 4 °C for 5 min. Collected biomass was freeze-dried overnight. Subsequently, it was homogenised with 2 mm glass beads for 15 s on a homogeniser. Then, 1.5 mL PBS buffer (pH 7.4) precooled to 4 ºC was added, and the sample was mixed with PBS for 5 s on the homogeniser. After being kept on ice for 60 min, the sample was centrifuged at 15,000× *g* and 4 °C for 5 min. Concentrations of phycobiliprotein and APC were quantified by measuring optical absorbance at 615, 652, and 720 nm with a spectrophotometer (UV-1800, Shimadzu Corporation, Kyoto, Japan) and were calculated as follows [31]:

(6) $$\mathrm{PC}\;\left[\mu g/\mathrm{mL}\right]=\left(\left(A_{615}-{\;A}_{720}\right)-0.474\times \left(A_{652}-{\;A}_{720}\right)\right)/5.34$$

(7) PC [μg/mg of biomass]= PC[μg/mL] / biomass concentration [mg/mL]

(8) $$\mathrm{APC}\;\left[\mu g/\mathrm{mL}\right]=\left(\left(A_{652}-{\;A}_{720}\right)-0.208\times \left(A_{615}-{\;A}_{720}\right)\right)/5.09$$

(9) Chla [μg/mg of biomass]= Chla[μg/mL] / biomass concentration[mg/mL]

### 4.4. Determination of FAMEs and Alkane

Analysis of fatty acid methyl esters (FAMEs) by in situ transesterification was conducted according to the procedure published by the National Renewable Energy Laboratory of the USA [32]. In brief, 5 mg biomass was transferred into a 1.5-mL GC vial. Subsequently, 20 μL of internal standard (C19:0), 200 μL of chloroform:methanol (2:1, *v*/*v*), and 300 μL of 0.6 M HCl:methanol were added. The tube was then incubated in a water bath at 85 °C for 1 h. After cooling to room temperature, 1 mL n-hexane was added to extract FAMEs and heptadecane. The tube was vortexed and allowed to stand undisturbed for 1 h. FAMEs and alkanes in n-hexane phase were analysed by gas chromatography (7890A, Agilent, Santa Clara, CA, USA) equipped with a flame ionisation detector (FID) and DB-23 column with a film thickness of 0.25 μm (30 m × 0.25 mm, length × internal diameter, Agilent Technologies, Santa Clara, CA, USA). The injection volume was 1 μL with a split ratio of 10:1. The temperature of the inlet and FID was 250 °C and 280 °C, respectively. Helium was used as a carrier gas at a constant flow rate of 1.0 mL·min^−1^. Programme of oven temperature: 50 °C for 1 min, 25 °C·min^−1^ up to 175 °C and hold for 0 min, 4 °C·min^−1^ up to 230 °C and hold for 5 min. FAMEs and heptadecane were identified by comparing elution time with a calibration standard. The concentration of FAMEs and heptadecane was determined by comparing with internal calibration curves made of 5 points with C19:0 as an internal standard (R^2^ > 0.998).

### 4.5. Lipidomic Analysis

For lipid extraction, 1–2 mg freeze-dried biomass was transferred into a 2 mL centrifuge tube. A total of 300 μL methanol was added, and the tube was vortexed for 20 s. Subsequently, 1000 μL MTBE (methyl tertiary butyl ether) was added, and the tube was vortexed for 5 min. Then, 250 μL deionised water was added, and the tube was vortexed for 30 s. The tube was left to stand for 10 min and centrifuged at 14,000 rpm for 2 min. The upper phase was transferred to a 2 mL vial and dried under nitrogen. Dried lipids were solubilised by 1 mL chloroform–methanol (1:1, *v*/*v*) and kept at −20 °C.

Separation of lipids was carried out by Dionex Ultimate 3000 UPLC equipped with ZORBAX Eclipse Plus RRHD C18 column (2.1 mm × 100 mm × 1.8 μm, Agilent Technologies, Santa Clara, CA, USA). In positive ion mode, oven temperature was 50 °C, and total flow rate was 0.300 mL/min. Solvent A was isopropanol–methanol–water (5:1:4, *v*/*v*/*v*) supplemented with 5 mM NH_4_OAc and 0.1% CH_3_COOH. Solvent B was isopropanol–water (99:1, *v*/*v*). Elution programme: 0–3 min, 0% B; 5 min, 20% B; 25 min, 30% B; 35 min, 95% B; 36 min, 95% B; 38 min, 0% B.

In negative ion mode, oven temperature was 45 °C. Total flow rate was 0.300 mL/min. Solvent A was acetonitrile–water (60:40, *v*/*v*) containing 10 mM NH_4_OAc. Solvent B was isopropanol–acetonitrile (90:10, *v*/*v*) containing 10 mM NH_4_OAc and 0.1% CH_3_COOH (*v*/*v*). Elution programme was: 0–1.5 min, 32% B; 4 min, 45% B; 5 min 52% B; 8 min 58% B; 11 min 66% B; 14 min 70% B; 18 min, 75% B; 21 min, 97% B; 25 min, 97% B; 25.1 min, 32% B; 30 min, 32% B.

Mass analysis was conducted by a quadrupole–orbitrap hybrid mass analyser (Q Exactive, Thermo Fisher Scientific, Wastham, MA, USA) equipped with HESI ion source. Both positive and negative mode was used. Resolution power for full scan and data-dependent MS/MS was 70,000 and 17,500, respectively. Spray voltage was 3.6 kV, capillary temperature was 300 °C, and auxiliary temperature was 370 °C. Sheath gas flow rate was 60 Arb; auxiliary gas flow rate was 25 Arb. MS^1^ *m*/*z* range was 220–1700.

Identification of lipids was achieved by searching the Lipidsearch 4.1.16 (Thermo Fisher Scientific, Waltham, MA, USA) database based on MS^2^, and was confirmed by comparing with MS^2^ of calibration standards. Quantitation was based on the peak area of MS^1^.

### 4.6. Determination of Carbohydrates and Elemental Analysis

The biomass was dissolved by a two-step sulphuric acid hydrolysis [33]. Firstly, 5 mg freeze-dried biomass and 50 μL 72 wt.% sulphuric acid was transferred into a 2 mL stoppered centrifuge tube. The tube was incubated at 30 °C for 60 min, being vortexed for 15 s every 15 min. Subsequently, 1.4 mL deionised water was added to dilute the concentration of sulphuric acid to 4 wt.%. The biomass was then hydrolysed by 4 wt.% sulphuric acids at 121 °C for 60 min in an autoclave (GF-DA, Zealway Instrument Inc., Wilmington, DE, USA). After being cooled to room temperature, the pH of the hydrolysate was adjusted to 5.0–7.0 by calcium carbonate. Glucose in the hydrolysate was analysed by high-performance liquid chromatography (Agilent 1260, Angilent Technologies, Santa Clara, CA, USA). Agilent Hi-Plex Ca column with a particle size of 8 μm (300 mm × 7.7 mm, length × internal diameter, Angilent Technologies, Santa Clara, CA, USA) was used to separate glucose with deionised water as mobile phase at a flow rate of 0.6 mL·min^−1^. The temperature of the oven and RI detector was 80 °C and 55 °C, respectively. The concentration of glucose was determined by comparing with an external calibration curve that consisted of five points (R^2^ > 0.9999).

Elemental analysis of C, H, N, and S was carried out on an elemental analyser (EA 2400 II, Perkin Elmer, Waltham, MA, USA) according to the manufacturer’s instructions. A total of 1–2 mg freeze-dried biomass was weighted for each analysis.

## Figures and Tables

**Table 1:** Effects of temperature and nitrogen supply on the growth rate and biomass yield of E542.

	Biomass –1st Stage (mgL^−1^)	Growth Rate μ –1st Stage (1 d^−1^)	Biomass –2nd Stage (mgL^−1^)	Growth Rate μ –2nd Stage (1 d^−1^)
35–48 h	0.117 ± 0.008	1.29 ± 0.11	0.265 ± 0.01	0.82 ± 0.07
35–NS	0.102 ± 0.005	1.32 ± 0.21	0.196 ± 0.009	0.65 ± 0.05
45–48 h	0.254 ± 0.003	2.26 ± 0.08	0.591 ± 0.012	0.84 ± 0.02
45–NS	0.253 ± 0.007	2.23 ± 0.17	0.605 ± 0.015	0.87 ± 0.03
55–48 h	0.244 ± 0.004	2.16 ± 0.13	0.678 ± 0.017	1.02 ± 0.07
55–NS	0.240 ± 0.004	2.19 ± 0.04	0.654 ± 0.018	1.00 ± 0.04

**Table 2:** Calculated parameters related to OJIP transients.

	F_*O*_	V_J_	V_I_	F_V_/F_M_
35–48 h	27,089 ± 186 ^1^	0.853 ± 0.016	0.794 ± 0.013	0.321 ± 0.007
45–48 h	48,923 ± 306	0.665 ± 0.007	0.811 ± 0.008	0.422 ± 0.008
55–48 h	44,738 ± 510	0.661 ± 0.008	0.835 ± 0.010	0.344 ± 0.006
35–NS	10,348 ± 181	0.992 ± 0.007	0.910 ± 0.006	0.180 ± 0.008
45–NS	14,796 ± 170	0.912 ± 0.007	0.956 ± 0.009	0.203 ± 0.006
55–NS	15,527 ± 421	0.853 ± 0.007	0.959 ± 0.006	0.181 ± 0.002

^1^ data are expressed with means ± standard error (n = 3).

**Table 3:** Content of various lipids and their fatty acid composition of *Thermosynechococcus* E542.

	35 °C	35 °C–NS	55 °C	55 °C–NS
MGDG content ^1^	17.8 ± 0.64 ^3^	12.2 ± 0.10	18.6 ± 1.4	13.8 ± 0.48
MGDG (16:0/16:1)	63.8 ± 2.2	47.1 ± 1.1	27.8 ± 0.9	21.2 ± 0.2
MGDG (16:0/18:1)	21.4 ± 0.2	35.7 ± 0.3	35.1 ± 1.3	34.6 ± 0.2
Others ^2^	14.8	17.2	37.1	44.2
DGDG content	26.7 ± 0.26	19.1 ± 1.0	25.1 ± 0.66	21.1 ± 1.1
DGDG (16:0/18:1)	17.4 ± 0.3	25.4 ± 1.6	32.0 ± 0.5	36.3 ± 1.3
DGDG (16:0/16:1)	60.1 ± 1.5	50.9 ± 4.3	25.0 ± 0.5	19.2 ± 0.3
DGDG (16:0/16:0)	6.2 ± 0.1	5.9 ± 0.5	20.1 ± 0.3	14.7 ± 0.1
Others	16.3	17.9	22.9	29.9
SQDG content	3.70 ± 0.11	1.75 ± 0.01	2.32 ± 0.05	1.42 ± 0.07
SQDG (16:0/16:0)	> 99			
PG content	2.20 ± 0.07	1.44 ± 0.01	1.58 ± 0.01	0.94 ± 0.05
PG (16:0/16:0)	8.3 ± 0.1	7.2 ± 0.1	19.1 ± 0.5	17.5 ± 0.1
PG (16:0/16:1)	49.9 ± 1.2	42.5 ± 0.1	61.9 ± 0.4	66.2 ± 0.2
PG (16:0/18:1)	41.8 ± 1.1	50.3 ± 0.2	0	0
PG (18:0/16:0)	0	0	19.0 ± 0.2	16.4 ± 0.2

^1^ MGDG content means the content of MGDG in dried biomass, the unit is mg/g. ^2^ Others mean lipid molecules with other fatty acid composition and this was calculated by subtracting the contents of given MGDG species from 100%. ^3^ Data are expressed as means ± standard error (n = 3).

**Table 4:** Elemental composition of different cultures.

	Carbon	Hydrogen	Nitrogen	Sulphur
35–48 h	39.40 ± 0.30 ^1^	7.28 ± 0.29	8.09 ± 0.04	0.68 ± 0.01
35–NS	38.77 ± 0.09	8.22 ± 0.29	3.92 ± 0.12	0.49 ± 0.01
45–48 h	42.55 ± 0.55	8.86 ± 0.25	9.36 ± 0.18	1.03 ± 0.11
45–NS	41.03 ± 0.76	10.20 ± 0.02	3.93 ± 0.09	0.66 ± 0.02
55–48 h	44.05 ± 1.30	11.07 ± 0.01	8.95 ± 0.33	1.02 ± 0.02
55–NS	42.92 ± 0.01	10.54 ± 0.11	4.28 ± 0.02	0.81 ± 0.14

^1^ Data are expressed as means ± standard error (n = 3).

## Data Availability

All data generated or analysed during this study are included in this published article.

## Supplementary Materials

The following are available online at https://www.mdpi.com/article/10.3390/plants10102101/s1, Table S1: title, Profiles of polar lipids in thermophilic cyanobacterium *Thermosynechococcus E542*.

## References

[1] Tang J., Liang Y., Jiang D., Li L., Luo Y., Shah M.M.R., Daroch M. (2018). Temperature-controlled Thermophilic Bacterial Communities in Hot Springs of Western Sichuan, China. BMC Microbiol..

[2] Chen P.-H., Liu H.-L., Chen Y.-J., Cheng H., Lin W.-L., Yeh C.-H., Chang C.-H. (2012). Enhancing CO_2_ Bio-mitigation by Genetic Engineering of Cyanobacteria. Energy Environ. Sci..

[3] Liang Y., Kaczmarek M.B., Kasprzak A.K., Tang J., Shah M.M.R., Jin P., Klepacz-Smolka A., Cheng J.J., Ledakowicz S., Daroch M. (2018). Thermosynechococcaceae as a Source of Thermostable C-phycocyanins: Properties and Molecular Insights. Algal Res. Biomass Biofuels Bioprod..

[4] Forchhammer K., Schwarz R. (2019). Nitrogen Chlorosis in Unicellular Cyanobacteria—A Developmental Program for Surviving Nitrogen Deprivation. Environ. Microbiol..

[5] Huner N.P.A., Oquist G., Sarhan F. (1998). Energy Balance and Acclimation to Light and Cold. Trends Plant Sci..

[6] Mackey K.R.M., Paytan A., Caldeira K., Grossman A.R., Moran D., McIlvin M., Saito M.A. (2013). Effect of Temperature on Photosynthesis and Growth in Marine *Synechococcus* spp.. Plant Physiol..

[7] Nikolova D., Weber D., Scholz M., Bald T., Scharsack J.P., Hippler M. (2017). Temperature-Induced Remodeling of the Photosynthetic Machinery Tunes Photosynthesis in the Thermophilic Alga Cyanidioschyzon merolae. Plant Physiol..

[8] Mikami K., Murata N. (2003). Membrane Fluidity and the Perception of Environmental Signals in Cyanobacteria and Plants. Prog. Lipid Res..

[9] Schwarz R., Forchhammer K. (2005). Acclimation of Unicellular Cyanobacteria to Macronutrient Deficiency: Emergence of a Complex Network of Cellular Responses. Microbiology.

[10] Maranon E., Lorenzo M.P., Cermeno P., Mourino-Carballido B. (2018). Nutrient Limitation Suppresses the Temperature Dependence of Phytoplankton Metabolic Rates. ISME J..

[11] Liang Y., Tang J., Luo Y., Kaczmarek M.B., Li X., Daroch M. (2019). *Thermosynechococcus* as a Thermophilic Photosynthetic Microbial Cell Factory for CO_2_ Utilisation. Bioresour. Technol..

[12] Tang J., Jiang D., Luo Y., Liang Y., Li L., Shah M.M.R., Daroch M. (2018). Potential New Genera of Cyanobacterial Strains Isolated from Thermal Springs of Western Sichuan, China. Algal Res. Biomass Biofuels Bioprod..

[13] Vonshak A., Novoplansky N. (2008). Acclimation to Low Temperature of Two Arthrospira platensis (Cyanobacteria) Strains Involves Down-regulation of PSII and Improved Resistance to Photoinhibition. J. Phycol..

[14] Tsimilli-Michael M., Stamatakis K., Papageorgiou G.C. (2009). Dark-to-light Transition in Synechococcus sp PCC 7942 Cells Studied by Fluorescence Kinetics Assesses Plastoquinone Redox Poise in the Dark and Photosystem II Fluorescence Component and Dynamics during State 2 to State 1 Transition. Photosynth. Res..

[15] Campbell D., Hurry V., Clarke A.K., Gustafsson P., Oquist G. (1998). Chlorophyll Fluorescence Analysis of Cyanobacterial Photosynthesis and Acclimation. Microbiol. Mol. Biol. Rev..

[16] Lu C., Vonshak A. (2002). Effects of Salinity Stress on Photosystem II Function in Cyanobacterial *Spirulina platensis* Cells. Physiol. Plant..

[17] Zhang T., Gong H., Wen X., Lu C. (2010). Salt Stress Induces a Decrease in Excitation Energy Transfer from Phycobilisomes to Photosystem II but an Increase to Photosystem I in the Cyanobacterium *Spirulina platensis*. J. Plant Physiol..

[18] Zhao B., Wang J., Gong H., Wen X., Ren H., Lu C. (2008). Effects of Heat Stress on PSII Photochemistry in a Cyanobacterium *Spirulina platensis*. Plant Sci..

[19] Wada H., Murata N. (1990). Temperature-induced changes in the Fatty-acid Composition of the Cyanobacterium, *Synechocystis* PCC6803. Plant Physiol..

[20] Berla B.M., Saha R., Maranas C.D., Pakrasi H.B. (2015). Cyanobacterial Alkanes Modulate Photosynthetic Cyclic Electron Flow to Assist Growth under Cold Stress. Sci. Rep..

[21] Shan Y., Liu Y., Yang L., Nie H., Shen S., Dong C., Bai Y., Sun Q., Zhao J., Liu H. (2016). Lipid Profiling of Cyanobacteria *Synechococcus* sp PCC 7002 Using Two-dimensional Liquid Chromatography with Quadrupole Time-of-flight Mass Spectrometry. J. Sep. Sci..

[22] Mavroudakis L., Valsami E.-A., Grafanaki S., Andreadaki T.-P., Ghanotakis D.F., Pergantis S.A. (2019). The Effect of Nitrogen Starvation on Membrane Lipids of *Synechocystis* sp. PCC 6803 Investigated by Using Easy Ambient Sonic-spray Ionization Mass Spectrometry. Biochim. Biophys. Acta-Biomembr..

[23] Pittera J., Jouhet J., Breton S., Garczarek L., Partensky F., Marechal E., Nguyen N.A., Dore H., Ratin M., Pitt F.D. (2018). Thermoacclimation and Genome Adaptation of the Membrane Lipidome in Marine *Synechococcus*. Environ. Microbiol..

[24] Collier J.L., Grossman A.R. (1994). A Small Polypeptide Triggers Complete Degradation of Light-harvesting Phycobiliproteins in Nutrient-deprived Cyanobacteria. EMBO J..

[25] Collier J.L., Grossman A.R. (1992). Chlorosis Induced by Nutrient Deprivation in *Synechococcus* sp Strain PCC-7942-Not All Bleaching is the Same. J. Bacteriol..

[26] Hasunuma T., Kikuyama F., Matsuda M., Aikawa S., Izumi Y., Kondo A. (2013). Dynamic Metabolic Profiling of Cyanobacterial Glycogen Biosynthesis under Conditions of Nitrate Depletion. J. Exp. Bot..

[27] Chow T.-J., Su H.-Y., Tsai T.-Y., Chou H.-H., Lee T.-M., Chang J.-S. (2015). Using Recombinant Cyanobacterium (*Synechococcus* elongatus) with Increased Carbohydrate Productivity as Feedstock for Bioethanol Production via Separate Hydrolysis and Fermentation Process. Bioresour. Technol..

[28] Li X., Li W., Zhai J., Wei H., Wang Q. (2019). Effect of Ammonium Nitrogen on Microalgal Growth, Biochemical Composition and Photosynthetic Performance in Mixotrophic Cultivation. Bioresour. Technol..

[29] Zavrel T., Sinetova M.A., Červený J. (2015). Measurement of Chlorophyll a and Carotenoids Concentration in Cyanobacteria. Bio-Protocol.

[30] Ritchie R.J. (2006). Consistent Sets of Spectrophotometric Chlorophyll Equations for Acetone, Methanol and Ethanol Solvents. Photosynth. Res..

[31] Zavrel T., Chmelik D., Sinetova M.A., Cerveny J. (2018). Spectrophotometric Determination of Phycobiliprotein Content in Cyanobacterium Synechocystis. Jove-J. Vis. Exp..

[32] Van Wychen S., Laurens L. (2013). Determination of Total Lipids as Fatty Acid Methyl Esters (FAME) by in Situ Transesterification. Contract.

[33] Sluiter A., Hames B., Ruiz R., Scarlata C., Sluiter B. (2008). Determination of Structural Carbohydrates and Lignin in Biomass.

