# Supplementary material for: Effects of Low Temperature, Nitrogen Starvation and Their Combination on the Photosynthesis and Metabolites of Thermosynechococcus E542: A Comparison Study
Source: Plants (Basel). 2021 Oct 4;10(10):2101. doi: 10.3390/plants10102101 (PMC8537721; doi:10.3390/plants10102101)
Supplement: Supplementary file 1 [file plants-10-02101-s001.zip › plants-1394522-supplementary.pdf]

Table S1 Profiles of polar lipids in in thermophilic cyanobacterium *Thermosynechococcus E542*

Lipid Species (Fatty acids)	Formula	Observed Mass	Calculated Mass
	$[M+CH_3COO]^-$		
**MGDG(16:0/18:1)**	$C_{45}H_{83}O_{12}$	815.5905	815.5890
**MGDG(16:0/16:1)**	$C_{43}H_{79}O_{12}$	787.5583	787.5577
**MGDG(16:0/16:0)**	$C_{43}H_{81}O_{12}$	789.5727	789.5734
**MGDG(16:0/19:1)**	$C_{46}H_{85}O_{12}$	829.6058	829.6047
**MGDG(17:0/18:1)**	$C_{46}H_{85}O_{12}$	829.6054	829.6047
**MGDG(16:0/17:1)**	$C_{44}H_{81}O_{12}$	801.5746	801.5734
**MGDG(16:0/18:0)**	$C_{45}H_{85}O_{12}$	817.6045	817.6047
**MGDG(16:1/18:1)**	$C_{45}H_{81}O_{12}$	813.5742	813.5734
**MGDG(18:0/18:1)**	$C_{47}H_{87}O_{12}$	843.6211	843.6203
**MGDG(16:0/17:0)**	$C_{44}H_{83}O_{12}$	803.5903	803.5890
**MGDG(14:0/16:1)**	$C_{41}H_{75}O_{12}$	759.5273	759.5264
**MGDG(14:1/16:0)**	$C_{41}H_{75}O_{12}$	759.5264	759.5264
**MGDG(18:1/18:1)**	$C_{47}H_{85}O_{12}$	841.6049	841.6047
**MGDG(14:0/16:0)**	$C_{41}H_{77}O_{12}$	761.5427	761.5421
**MGDG(16:1/16:1)**	$C_{43}H_{77}O_{12}$	785.5427	785.5421
**MGDG(16:1/19:1)**	$C_{46}H_{83}O_{12}$	827.5899	827.5890
**MGDG(16:0/20:0)**	$C_{47}H_{89}O_{12}$	845.6377	845.6360
**MGDG(16:1/17:1)**	$C_{44}H_{79}O_{12}$	799.5590	799.5577
**MGDG(16:0/18:2)**	$C_{45}H_{81}O_{12}$	813.5745	813.5734
**MGDG(12:0/16:0)**	$C_{39}H_{73}O_{12}$	733.5108	733.5108
**MGDG(16:0/19:0)**	$C_{46}H_{87}O_{12}$	831.6220	831.6203
**MGDG(18:0/19:1)**	$C_{48}H_{89}O_{12}$	857.6384	857.6360
**MGDG(18:1/19:1)**	$C_{48}H_{87}O_{12}$	855.6200	855.6203
**MGDG(15:0/16:1)**	$C_{42}H_{77}O_{12}$	773.5404	773.5421

**MGDG(14:0/17:1)**	$C_{42} H_{77} O_{12}$	773.5430	773.5421
**MGDG(17:1/18:1)**	$C_{46} H_{83} O_{12}$	827.5923	827.5890
**MGDG(17:1/19:1)**	$C_{47} H_{85} O_{12}$	841.6069	841.6047
**MGDG(12:0/16:1)**	$C_{39} H_{71} O_{12}$	731.4958	731.4951
**MGDG(16:0/16:2)**	$C_{43} H_{77} O_{12}$	785.5433	785.5421
	$[M+CH3COO]^-$		
**DGDG(16:0/18:1)**	$C_{51} H_{93} O_{17}$	977.6424	977.6418
**DGDG(16:0/16:1)**	$C_{49} H_{89} O_{17}$	949.6112	949.6105
**DGDG(16:0/16:0)**	$C_{49} H_{91} O_{17}$	951.6271	951.6262
**DGDG(16:0/19:1)**	$C_{52} H_{95} O_{17}$	991.6585	991.6575
**DGDG(17:0/18:1)**	$C_{52} H_{95} O_{17}$	991.6587	991.6575
**DGDG(16:0/17:1)**	$C_{50} H_{91} O_{17}$	963.6274	963.6262
**DGDG(16:0/18:0)**	$C_{51} H_{95} O_{17}$	979.6575	979.6575
**DGDG(16:1/18:1)**	$C_{51} H_{91} O_{17}$	975.6263	975.6262
**DGDG(14:0/16:0)**	$C_{47} H_{87} O_{17}$	923.5964	923.5949
**DGDG(16:0/17:0)**	$C_{50} H_{93} O_{17}$	965.6440	965.6418
**DGDG(18:1/18:1)**	$C_{53} H_{95} O_{17}$	1003.6593	1003.6575
**DGDG(18:0/18:1)**	$C_{53} H_{97} O_{17}$	1005.6746	1005.6731
**DGDG(16:1/19:1)**	$C_{52} H_{93} O_{17}$	989.6419	989.6418
**DGDG(17:1/18:1)**	$C_{52} H_{93} O_{17}$	989.6436	989.6418
**DGDG(16:1/16:1)**	$C_{49} H_{87} O_{17}$	947.5948	947.5949
**DGDG(14:0/16:1)**	$C_{47} H_{85} O_{17}$	921.5808	921.5792
**DGDG(14:1/16:0)**	$C_{47} H_{85} O_{17}$	921.5807	921.5792
**DGDG(16:0/18:2)**	$C_{51} H_{91} O_{17}$	975.6266	975.6262
**DGDG(16:1/17:1)**	$C_{50} H_{89} O_{17}$	961.6113	961.6105
**DGDG(12:0/16:0)**	$C_{45} H_{83} O_{17}$	895.5645	895.5636
	$[M-H]^-$		
**SQDG(16:0/16:0)**	$C_{41} H_{77} O_{12} S_1$	793.5141	793.5141
**SQDG(16:1/16:1)**	C41 H73 O12 S1	789.4842	789.4828

**SQDG(18:0/18:0)**	$C_{45} H_{85} O_{12} S_1$	849.5778	849.5767
	$[M+NH4]^+$		
**PG(16:0/16:0)**	$C_{38} H_{79} O_{10} N_1 P_1$	740.5464	740.5436
**PG(16:0/18:1)**	$C_{40} H_{81} O_{10} N_1 P_1$	766.5617	766.5593
**PG(18:0/16:0)**	$C_{40} H_{83} O_{10} N_1 P_1$	768.5747	768.5749
**PG(16:0/16:1)**	$C_{38} H_{77} O_{10} N_1 P_1$	738.5303	738.5280
